# Improving resolution of dynamic communities in human brain networks through targeted node removal

**DOI:** 10.1371/journal.pone.0187715

**Published:** 2017-12-20

**Authors:** Kimberly J. Schlesinger, Benjamin O. Turner, Scott T. Grafton, Michael B. Miller, Jean M. Carlson

**Affiliations:** 1 Department of Physics, University of California Santa Barbara, Santa Barbara, California, United States of America; 2 Department of Psychological and Brain Sciences, University of California Santa Barbara, Santa Barbara, California, United States of America; Ghent University, BELGIUM

## Abstract

Current approaches to dynamic community detection in complex networks can fail to identify multi-scale community structure, or to resolve key features of community dynamics. We propose a targeted node removal technique to improve the resolution of community detection. Using synthetic oscillator networks with well-defined “ground truth” communities, we quantify the community detection performance of a common modularity maximization algorithm. We show that the performance of the algorithm on communities of a given size deteriorates when these communities are embedded in multi-scale networks with communities of different sizes, compared to the performance in a single-scale network. We demonstrate that targeted node removal during community detection improves performance on multi-scale networks, particularly when removing the most functionally cohesive nodes. Applying this approach to network neuroscience, we compare dynamic functional brain networks derived from fMRI data taken during both repetitive single-task and varied multi-task experiments. After the removal of regions in visual cortex, the most coherent functional brain area during the tasks, community detection is better able to resolve known functional brain systems into communities. In addition, node removal enables the algorithm to distinguish clear differences in brain network dynamics between these experiments, revealing task-switching behavior that was not identified with the visual regions present in the network. These results indicate that targeted node removal can improve spatial and temporal resolution in community detection, and they demonstrate a promising approach for comparison of network dynamics between neuroscientific data sets with different resolution parameters.

## Introduction

In the study of complex networks, dynamic community detection is a method for identifying highly intraconnected clusters of nodes within a network and quantifying how these clusters change over time. In many cases, the identified clusters, or communities, correspond to modules that perform an identifiable functional or structural role, thus giving insight into the composition and organization of a network [1]. Detecting temporally changing clusters enables an analysis of how the roles of these modules evolve, and how the network reorganizes itself on various timescales [2]. In network neuroscience, communities highlight the organization of interacting neurons or brain regions [3]. The application of these methods to large-scale MRI-based structural and functional brain networks has identified broad organizational similarities shared between distinct brains [4–6], and quantified changes in brain dynamics across cognitive states, demographic measures, and time [7–9].

Although dynamic community detection has contributed to a more complete understanding of the brain, challenges remain in applying it to large-scale functional brain networks for predictive and diagnostic purposes [3]. Functional modules exist at a variety of sizes and temporal ranges, and community methods rely on parameter choices to resolve clusters at scales relevant to a specific question or investigation [3, 7, 10]. In the absence of clear “ground truth” knowledge against which to evaluate methods, strategies for choosing these parameters have varied widely and are often based upon the statistical robustness of results [3, 11]. Thus, groups of brain regions that are especially strongly correlated may dominate the identified community structure, obscuring the resolution of other functional modules or dynamic properties. For example, during tasks with a visual component, brain regions in the visual cortex form a highly coherent community that strongly affects the selection of resolution parameters. This may prevent a community detection algorithm from resolving modules that perform other cognitive functions during the task. An incomplete understanding of the effects of these parameter choices also complicates comparison across analyses with different parameters.

Here, we introduce an approach for targeted removal of network nodes to improve resolution in dynamic community detection. We demonstrate the approach in a synthetic network of oscillators, in which we precisely quantify detection performance by comparing to well-defined “ground truth” communities. We show that the presence of multi-scale organization inhibits community detection in these oscillator networks. We further demonstrate that removal of targeted subsets of nodes during community detection improves the resolution of communities among the remaining nodes.

We demonstrate the utility of targeted node removal in neuroscience applications by applying this method to dynamic functional brain networks from two distinct fMRI experiments. One features repetition of a single cognitive task and the other encompasses the performance of multiple different tasks. Commonly used community detection methods fail to resolve the substantial dynamic differences between these single- and multi-task data sets. However, with targeted removal of the regions in visual cortex, which contains the most functionally coherent brain regions, community detection reveals clear differences in the dynamic network properties of the two data sets on a population level. In addition, removal of visual regions improves the ability of these methods to spatially resolve groups of brain regions known to be functionally similar, especially in the multi-task data set. These results show that targeted node removal can both improve resolution of community dynamics in a single data set, and also enable comparison of community structures across data sets.

### Background and motivation

Community detection algorithms aim to use the connectivity information of a network to identify a network partition, or a division of the network nodes into clusters, such that each cluster is composed of nodes strongly connected within the cluster and weakly connected to other clusters. Unsupervised community detection methods often uncover useful or intuitive groupings: they can extract official affiliations based on interactions in human social networks [12]; identify similar regions in a field of view to aid image processing and compression [1, 13, 14]; and classify biochemical species based on their dynamics within metabolic networks [15].

Developing practical applications of community detection for specific networked systems requires choosing various context-dependent model parameters. In network neuroscience, this choice is especially challenging. Very little is known about fundamental principles that underlie the dynamic organization of large-scale brain regions, and a “ground truth” benchmark for functional communities is not well-understood in a network context. In current research, evaluation of community “correctness” is largely based on correspondence with anatomy, pre-existing knowledge of functional roles, or statistical analysis of community robustness [3, 4]. There is therefore little consensus about the best methods for imposing constraints on community detection algorithms and for evaluating results.

Additionally, the human brain has inherently multi-scale organization. This includes hierarchical modularity (communities within communities), interlocking communities of varying sizes, and communities that dynamically reconfigure over time [2, 5, 7, 16, 17]. Furthermore, brain dynamics related to a given experimental condition cannot be easily isolated. During an MRI scanning session, many brain circuits of multiple sizes and strengths, both related and unrelated to the phenomenon under study, are simultaneously active. Brain regions with particularly strong or consistently coherent activity, such as those in sensory or motor cortices during recruitment of those functions, may dominate the community structure detected in a data-driven analysis, masking the dynamic properties of other brain areas more relevant to the study.

The noisy, multi-scale, and dynamic nature of brain dynamics is not necessarily suited to common community detection algorithms. The “modularity maximization” method for community detection is widely used, computationally efficient, easily implemented, and natural to extend to weighted, signed, and dynamic networks [3, 18]. However, it has also been shown to have an inherent resolution limit [19] and requires the choice of parameters defining spatial and temporal scales [2, 3, 11]. Several heuristics have been developed to ensure that the detected communities represent organization at an informative scale. These typically involve either exploring many possible scales, or choosing communities at a scale that gives the most consistent partitions [3, 10, 11]. Such methods have enabled reasonable statistical confidence in results from a single individual or data set. However, approaches for comparing results of different resolutions between different individuals or data sets remain elusive.

### Strategy

This paper demonstrates that targeted removal of network nodes during community detection can be leveraged to improve the resolution of communities on multiple scales, and to aid in the principled comparison of community structure across data sets. Specifically, we study the effect of removing subsets of nodes with particularly strongly connected or coherent dynamics, as determined by observation or through knowledge of their functional role. For example, sensory or motor regions in large-scale functional brain networks are often highly coherent during tasks that recruit these functions, which may hinder the resolution of the dynamics of other network nodes in community detection.

**Fig 1:**
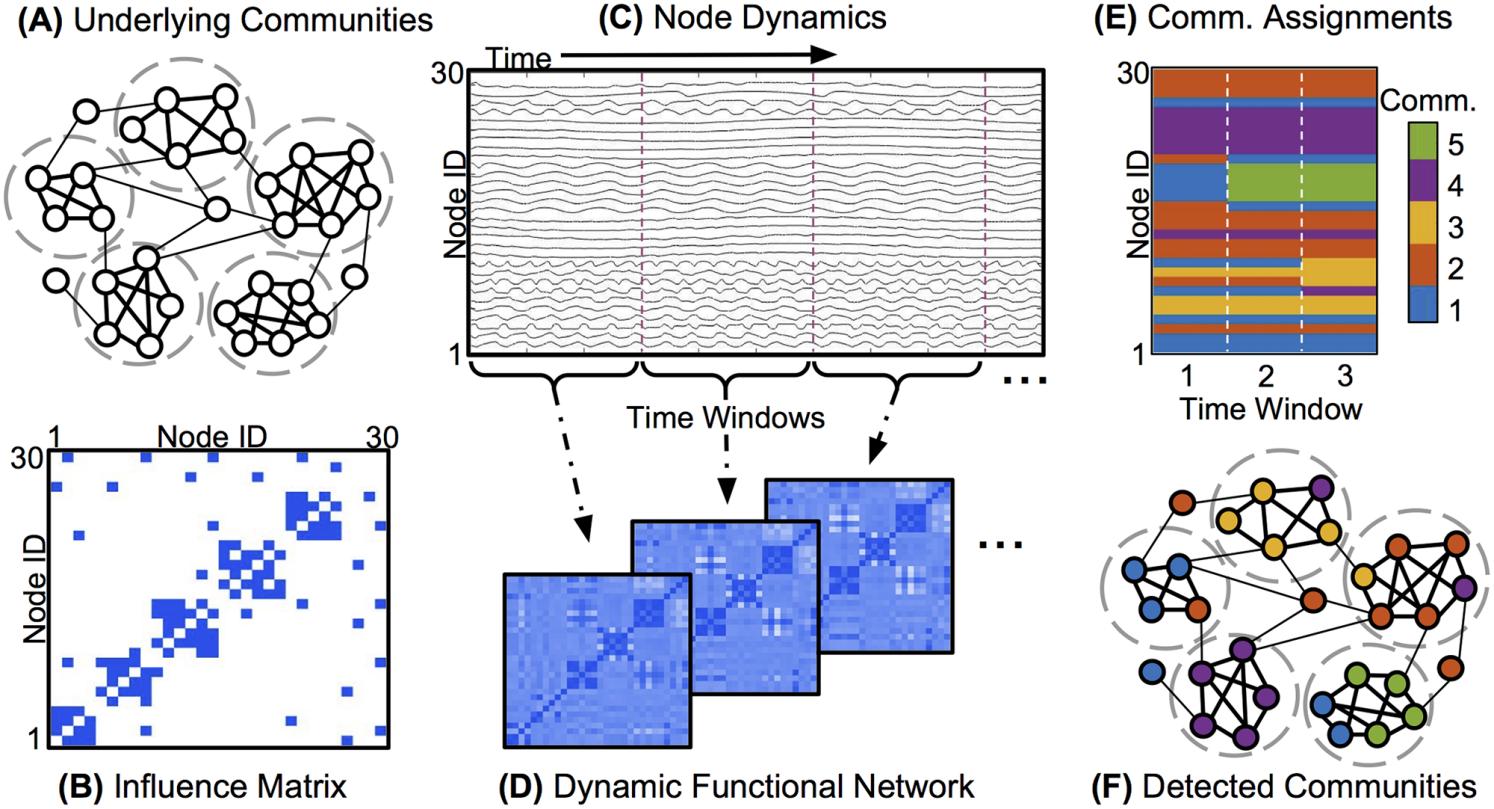
Schematic: Community detection on dynamic networks. A: Representation of the nodes and edges of a modular network, with dashed circles indicating the underlying communities. B: Binary *influence matrix* corresponding to this network, in which blue entries indicate a direct influence between node pairs and white entries indicate no direct influence. C: An example of node dynamics produced by this network, where each node is modeled as an oscillator with an intrinsic frequency, and nodes are influenced by their neighbors according to Eq 5. The evolving phases of successive nodes are stacked along the y-axis. D: Synchronization matrices representing the dynamic functional network derived from the time series in C. Each sequential matrix shows the synchronization between node pairs, averaged across the corresponding time window. E: Dynamic communities detected in the dynamic functional network in D. Each node is assigned to a single community (denoted by color) in each time window, and may switch community assignments between time windows. F: Comparison of dynamic community assignments from the third time window in E (denoted by color) to the underlying communities of influence from A (denoted by dashed circles).

**Fig 2:**
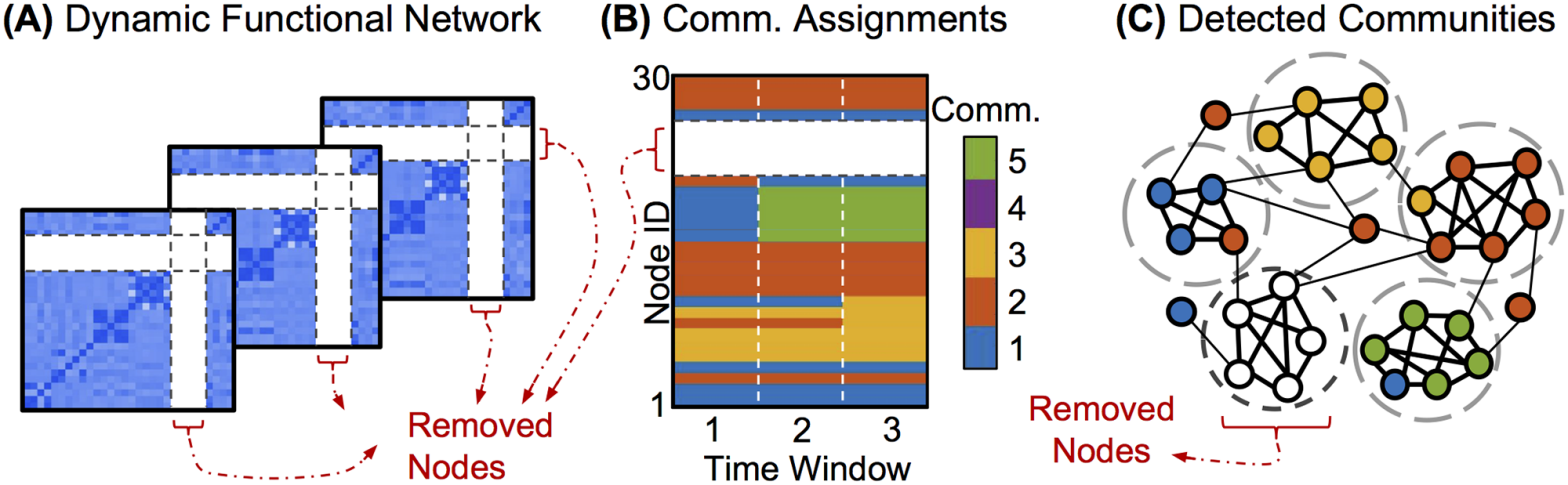
Schematic: Targeted removal of nodes for community detection. A: From the measured dynamics of the modular network in Fig 1, new dynamic functional network matrices are computed, with the functionally cohesive community removed (nodes 22-26). B: This truncated functional network is then used to detect dynamic communities, producing assignments for all remaining nodes. C: The resulting community structure from the third time window, denoted by color, provides a clearer identification of the underlying communities than the community structure detected with all nodes taken into account (Fig 1F).

We first illustrate the node removal approach in synthetic oscillator networks, in which results are evaluated based on a clear underlying “ground truth” network. A schematic of the approach to community detection in these networks is shown in Figs 1 and 2. We designate an underlying adjacency matrix (Fig 1B) for each synthetic network (Fig 1A), organized into modules that we define. This matrix, which we refer to as the “influence matrix,” represents the causal influences between oscillators that drive the synchronization dynamics of the network. This influence matrix serves as a rough analogy to the set of underlying anatomical connections and/or functional influences that produce the dynamics of brain activity measured with fMRI. It is not meant to represent the physical structure or architecture of the brain, but rather the basic functional organization that underlies the observed neural activity, and which we elucidate with community detection.

Once an influence matrix is defined for a synthetic oscillator network, we simulate the resulting network dynamics (Fig 1C), which we then “measure” by computing the synchronization between node pairs across a set of time windows (Fig 1D). Node pairs within the same underlying community tend to synchronize more closely than pairs in different communities. We produce a community partition (Fig 1E) from the dynamic network formed by the synchronization measurements, by applying a modularity-maximization community detection algorithm to this synchronization network [2, 18]. We use true and false positive rates to quantify the extent to which this partition matches the original underlying communities (Fig 1F). Finally, we apply community detection again, this time using information from only a subset of nodes in the synchronization matrices, and compare the results to community detection on the full network (Fig 2). Note that when removing nodes, the dynamics are still simulated for the full underlying network. Nodes are removed only from the dynamic synchronization networks (shown in Figs 1D and 2A) before the community detection algorithm is applied.

We find that in the precisely controlled oscillator networks, removal of certain subsets of nodes during community detection can improve the resolution of communities among the remaining nodes. We then apply our methods to functional human brain networks using two data sets, corresponding to two separate functional MRI experiments with different temporal task structures. In these brain networks, removal of the strongly correlated visual cortex from the dynamic adjacency matrix allows for better resolution of the differences between community structure during different cognitive tasks.

## Materials and methods

We first describe the method of modularity maximization used to detect dynamic communities throughout this paper, including the algorithms used to perform the maximization and find a stable community structure. We focus on this technique due to its widespread use for community detection in network neuroscience [3], which stems from its clear conceptual definition, the ease of adapting it to weighted and dynamic networks [20], and the existence of multiple computationally efficient implementations [2, 18]. We also define the metrics used to quantify community properties and assess detection performance. Finally, we introduce the two types of networks to which we apply these community detection methods: synthetic networks of nonlinear Kuramoto oscillators, with dynamics simulated *in silico*; and dynamic networks of human brain function, derived from fMRI measurements of brain activity while participants performed different sets of cognitive tasks.

### Community detection methods

We consider a network of *N* nodes connected by weighted, unsigned edges. Each edge may take on a different positive, real-valued weight in each of *T* time windows; *A*_*ijt*_ denotes the weight of the edge between node *i* and node *j* in time window *t*. In order to identify the optimal partition of nodes into modular communities, we seek the partition that maximizes the multislice modularity,

$$\begin{array}{c} Q=\frac{1}{2\mu }\sum_{ijtr}\{(A_{ijt}-\gamma P_{ijr})\delta_{tr}+\omega \delta_{ij}\}\delta (g_{it},g_{jr}), \end{array}$$ (1)

which indicates the quality of the modular structure of a partition in comparison to a randomized “null” network, *P*_*ijt*_ [2].

This quantity considers all node pairs *i*, *j* and all time window pairs *t*, *r* in which the community assignment of node *i* in window *t* (*g*_*it*_) is the same as the community assignment of node *j* in window *r* (*g*_*jr*_). For each node pair assigned to the same community in the same time window, the first term in the brackets provides a positive contribution to *Q* if the actual edge weight between the pair compares favorably to that in the null model. A spatial resolution factor *γ* determines the relative weight given to the null model. For each node *j* and each pair of time windows, the second term provides a positive contribution of *ω* to *Q* when *j* is assigned to the same community in both time windows [2]. Thus, maximizing *Q* favors network partitions in which the weights between nodes in the same community are greater than those expected in the null model, as well as those that group more nodes in the same community as themselves across multiple time windows.

In this study we use the Newman-Girvan null model, which treats edge weights as randomly distributed within each time window while preserving the node degree distribution [12]. We maximize *Q* over network partitions with a Louvain-like locally greedy algorithm implemented in MATLAB [2, 18]. Due to the stochasticity of the algorithm and the expected high degeneracy of solutions near the maximum value of *Q*, we use a community consensus procedure to distill a statistically representative partition from an ensemble of 100 solutions for each network (for more details, see [11] and [8]).

#### Resolution parameters

The value of the multislice modularity *Q* depends upon the values of *γ*, a spatial resolution parameter, and *ω*, a temporal resolution parameter. These parameters control the relative weight given to the null model in calculating *Q*, and thus alter the spatial and temporal scales at which communities will be found. A higher value of *γ* gives more weight to the null model, requiring much stronger connections to be present within a subset of nodes before they are counted as sufficiently different from the null model to constitute a community. A lower value of *γ* requires less connectivity within a potential community before it is deemed to be significant. Thus, lower values encourage few and large communities (*γ* = 0 will always return a single community containing every connected network node), while higher *γ* values tend to produce more, smaller communities.

In terms of temporal resolution, higher *ω* values place a greater value on maintaining the community assignment of a node across time windows of the network, and tend to produce partitions in which the node assignments are more similar to each other across time windows. Lower *ω* values give temporally consistent node assignments less weight, and in the case where *ω* = 0, the dynamic community detection task becomes equivalent to performing static community detection on each time window separately.

#### Metrics for community structure and detection performance

For a given community partition, the *community number* is defined as the total number of distinct community assignments given to the network nodes. The community number is always between 1 (all nodes in the same community) and *N* (each node in a different community) for modularity maximization methods.

The *flexibility* of a node in a dynamic network is defined as the number of times that node switches communities between adjacent time windows, normalized by the total possible number of switches:

$$\begin{array}{c} f\left(i\right)=\frac{1}{T-1}\sum_{t=1}^{T-1}[1-\delta (g_{it},g_{i(t+1)})]. \end{array}$$ (2)

Here, *T* is the total number of time windows; *δ*(*g*_*it*_, *g*_*it*′_) equals 1 if node *i* is assigned to the same community in slice *t* and slice *t*′, and 0 otherwise. A node with high flexibility changes communities in every or nearly every time window and has a flexibility at or near 1, while a node with low flexibility may remain in the same community in all windows and have a flexibility of 0. For example, in the schematic representation of community assignments for a dynamic network in Fig 1E, nodes 1 through 6 remain in the same community across all three time windows and have a flexibility of 0. However, node 7 switches from community 1 to community 4 between the last two time windows, and thus has a higher flexibility.

The *whole-network flexibility* is defined as the mean flexibility over all network nodes, and can be used to compare network dynamics under different conditions.

In cases with available “ground truth” communities, we use *true* and *false positive rates* to quantify community detection performance. We define these in terms of node pair co-assignments, comparing whether each node pair is truly in the same community (a “true” co-assignment) or not, and whether the node pair is “identified” as belonging to the same community by the algorithm or not. These quantities are defined as follows:

$$\begin{array}{c} \text{true}\;\text{positive}\;\text{rate}\;\left(\text{TPR}\right)=\frac{tp}{tp+fn}, \end{array}$$ (3)

$$\begin{array}{c} \text{false}\;\text{positive}\;\text{rate}\;\left(\text{FPR}\right)=\frac{fp}{fp+tn}. \end{array}$$ (4)

Here, *tp* indicates the number of true positives—i.e., node pair co-assignments both existent in the underlying influence matrix (“true”) and identified by the algorithm (“identified”). Similarly, *fp* indicates the number of false positives (“identified” co-assignments which are not “true”); *fn* the number of false negatives (“non-identified” co-assignments that are “true” in the influence matrix); and *tn* the number of true negatives (“non-identified” co-assignments which are also not “true”). Here, we compute the *TPR* and *FPR* separately for each network instance in an ensemble.

For each network, we summarize the performance of the community detection algorithm over an entire ensemble of networks with a *detection probability matrix* *D*. Each entry *D*_*ij*_ gives the fraction of network instances in which nodes *i* and *j* are identified as belonging to the same community. Perfect detection performance over all instances would result in *D*_*ij*_ = 1 for all *i*, *j* pairs in the same underlying community, and *D*_*ij*_ = 0 for all other pairs. *D*_*ij*_ < 1 (for *i* and *j* in the same underlying community) or *D*_*ij*_ > 0 (for *i* and *j* in different underlying communities) indicate that there are network instances in which the node pair co-assignment was incorrectly identified. When compared to the underlying influence matrix, *D* specifies which particular node pairs were correctly or incorrectly identified, contributing to the overall true and false positive rates.

**Fig 3:**
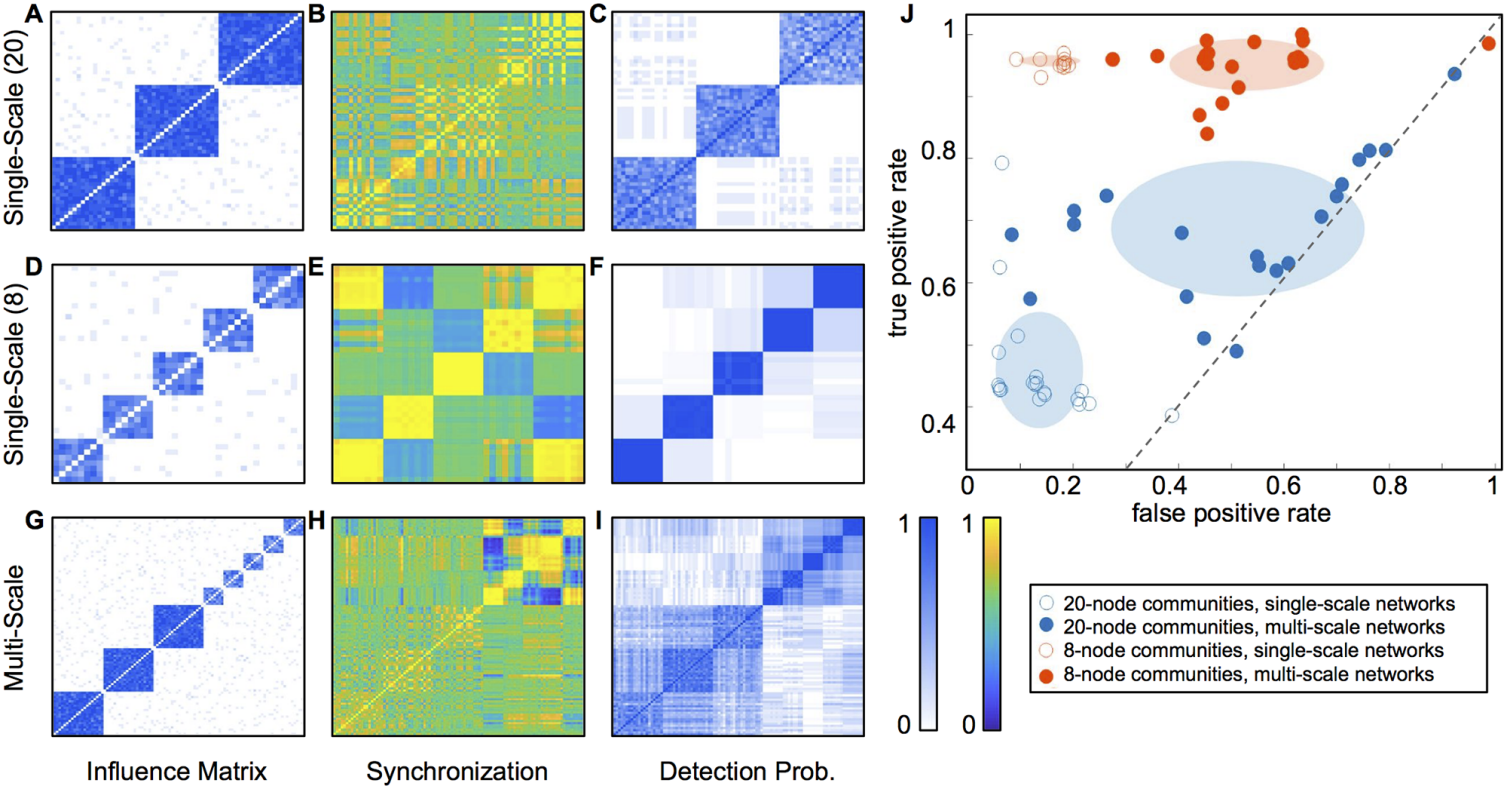
Community detection in single-scale networks outperforms multi-scale networks. Binary underlying influence matrices, examples of synchronization dynamics, and detection probability matrices for single-scale networks of 20-node communities (A-C), single-scale networks of 8-node communities (D-F), and multi-scale networks with both community sizes (G-I). Each influence matrix is averaged over an ensemble of 20 matrices generated with the same connection probabilities, and each detection probability matrix is averaged over the results from the same ensemble. Panel J summarizes the results, with each dot indicating the true and false positive rates for one network instance from an ensemble. Shaded ellipses highlight the ensemble shapes, with the ellipse centers at the ensemble means and the ellipse axes corresponding to the standard deviations. Unfilled dots represent results for single-scale networks with one community size only, with 20-node communities in blue and 8-node communities in orange. Filled dots represent results for 20-node communities (blue) and 8-node communities (orange) embedded in the multi-scale network of panels G-I. The community detection algorithm shows low false positive rates on single-scale networks, but substantially higher false positive rates on the same communities within multi-scale networks. This indicates that communities of the same size are more difficult to resolve when embedded in multi-scale networks.

**Fig 4:**
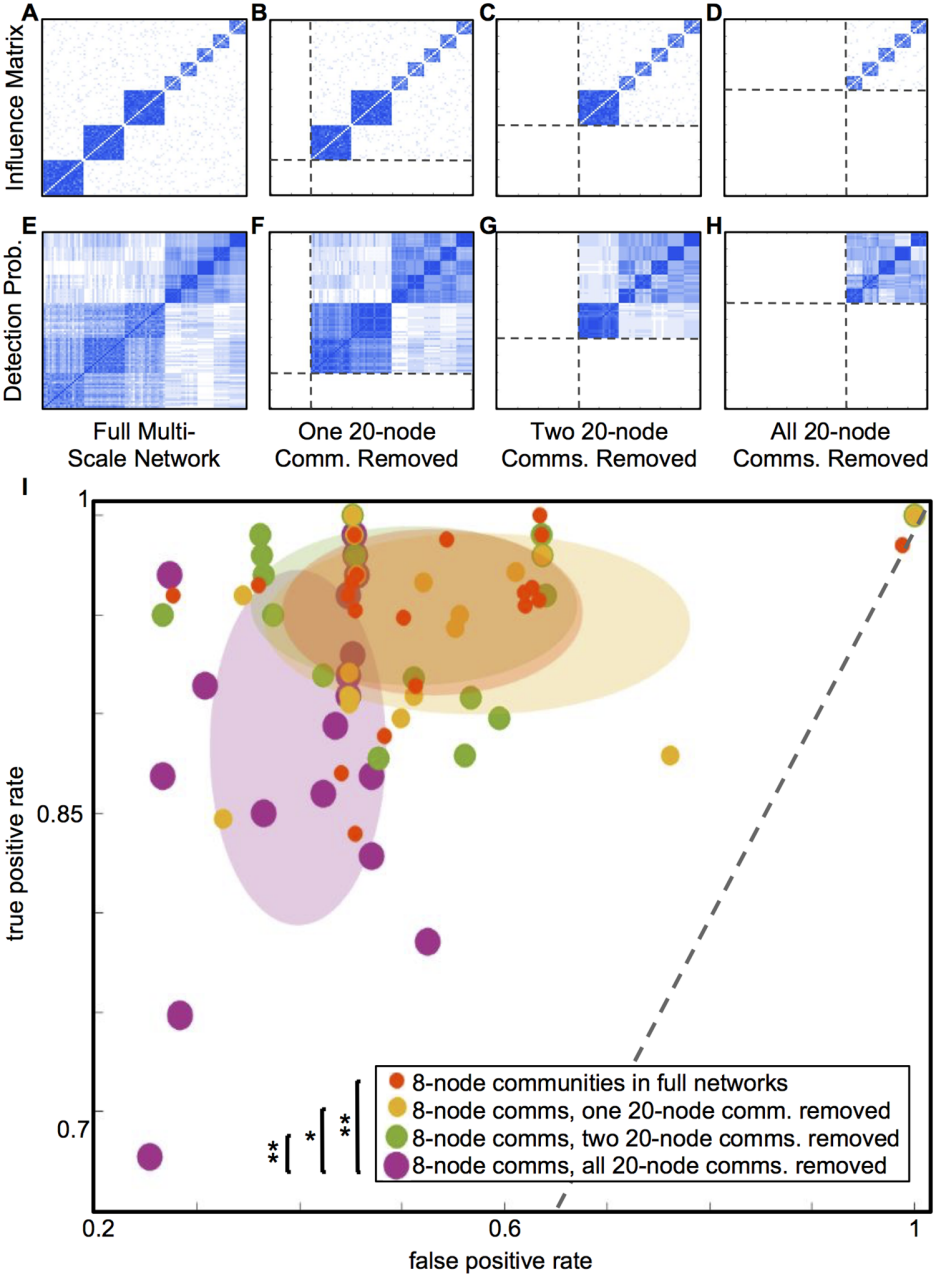
Removing larger communities slightly affects detection of smaller communities. Removal of larger, less coherent communities during community detection slightly affects detection of smaller, more coherent communities. The figure shows average influence matrices (A-D) and detection probability matrices (E-H) for the full multi-scale networks, as well as the same networks with one, two, and three 20-node communities removed. Panel I summarizes the results, plotting the true versus false positive rates for the detection of co-assignments of pairs of nodes in 8-node communities. Each dot represents results for one instance of an ensemble of 20 networks. A shaded ellipse highlights each ensemble, with the center at the ensemble mean and the axes corresponding to the standard deviations. Successive removal of larger communities (yellow and red distributions) does not make a significant difference in the detection of smaller communities, compared to in the full multi-scale network (orange). Only with all 20-node communities removed (purple) does the distribution of results change. Single stars by the figure legend indicate pairs of distributions that differ significantly in false positives only; double stars indicate a significant difference both true and false positives.

**Fig 5:**
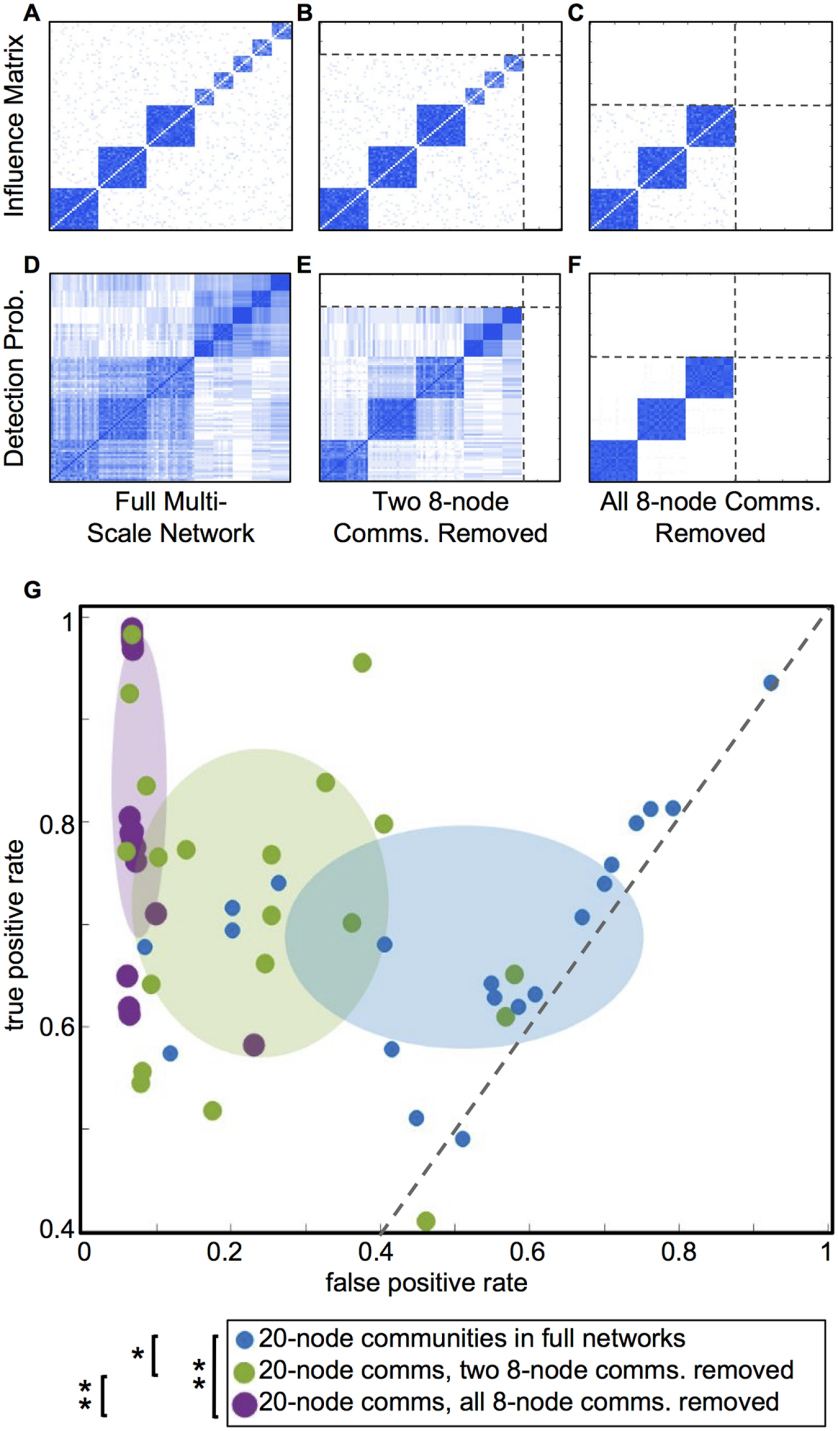
Removing smaller, more coherent communities improves detection of larger, less coherent communities. Average influence matrices (A-C) and detection probability matrices (D-F) for the full multi-scale network, as well as the same network with two and five 8-node communities removed. Panel G summarizes the results, plotting the true versus false positive rates for the detection of co-assignments of pairs of nodes in 20-node communities. Each dot represents results for one instance of an ensemble of 20 networks. A shaded ellipse highlights each ensemble, with the center at the ensemble mean and the axes corresponding to the standard deviations. Full multi-scale networks are shown in blue; networks with two small communities removed in green; and networks with all five small communities removed in purple. Each successive removal of more 8-node communities substantially improves detection performance, enabling the algorithm to distinguish the 20-node communities from each other. Single stars by the figure legend indicate pairs of distributions that differ significantly in false positives only; double stars indicate a significant difference both true and false positives.

We choose *TPR*, *FPR*, and the detection probability matrix to evaluate the community detection performance in order to shed light on the differences between different types of detection failures. Separating true positive rate and false positive rate—as opposed to using a measure such as Rand index which counts all correctly identified pair associations (both true positives and true negatives) together—allows easy visualization of the difference between community partitions that tend to combine underlying communities together (high false positive rate) and those that tend to split them apart (low true positive rate). Plotting *TPR* against *FPR*, as in Figs 3, 4 and 5, provides a visual interpretation of the performance of the detection algorithm compared to its expected random-chance performance (where *TPR* = *FPR*), and perfect delineation of communities (the upper left-hand corner, where *TPR* = 1 and *FPR* = 0).

### Synthetic networks of Kuramoto oscillators

We apply these community detection methods and techniques for improving resolution of community detection to synthetic networks of Kuramoto oscillators, in which the underlying influence matrix can be precisely controlled.

Following [21], we define each of the *N* nodes of a Kuramoto network as an oscillator indexed by *i* (*i* = 1, 2, …, *N*), whose time-dependent internal state is given by the angle *θ*_*i*_(*t*) ∈ [0, 2*π*). The state of each oscillator *i* evolves according to

$$\begin{array}{c} \frac{d\theta_{i}}{dt}=\omega_{i}+\sum_{j}\kappa C_{ij\;}\text{sin}\left(\theta_{j}-\theta_{i}\right), \end{array}$$ (5)

where *ω*_*i*_ is the intrinsic frequency and the second term describes the influence of other oscillators in the network. Interactions between oscillators are governed by a scaling factor *κ* and a time-independent, *N* x *N* binary matrix *C*, where *C*_*ij*_ = 1 denotes a *direct* influence between oscillators *i* and *j*, such that their dynamics will tend to synchronize over time. *C*_*ij*_ = 0 denotes no direct influence between *i* and *j*, although there may still be synchronization between them as a result of indirect influence or by chance. We refer to *C* as the *influence matrix* of the network.

We design *C* to have a modular structure, consisting of a number of communities of influence. Any pair of nodes in the same community have a directly influential relationship with probability *P*_*in*_, while a pair of nodes in two different communities is directly related with probability *P*_*out*_.

We examine two distinct network types determined by their underlying influence structure: single-scale and multi-scale. *Single-scale* networks are composed of communities that are all the same size. We consider single-scale communities of either 20 nodes or 8 nodes each. *Multi-scale* networks contain a collection of communities of different sizes. We focus on a multi-scale network of *N* = 100 nodes, consisting of eight total communities, with three 20-node communities and five 8-node communities. For all networks, we use *P*_*in*_ = 0.9 for 20-node communities, *P*_*in*_ = 0.7 for 8-node communities, and *P*_*out*_ = 0.01 for all out-of-community connections.

After determining an influence matrix *C*, we initiate each network with normally distributed intrinsic oscillator frequencies (*σ* = 1) and solve the network dynamics numerically with *κ* = 0.2. The dynamics of such a network begin in a random state, and the oscillators approach a steady state of partial synchronization after a short transient period. We quantify the observed functional dynamics of the network by computing the time-dependent *synchronization* [21]

$$\begin{array}{c} \phi_{ij}\left(t\right)=\left|\text{cos}\left(\theta_{i}\left(t\right)-\theta_{j}\left(t\right)\right)\right|. \end{array}$$ (6)

We then apply the community detection method to these synchronization dynamics, represented in the adjacency matrix *Φ*. The entries *Φ*_*ijr*_ are determined by averaging the synchronization *ϕ*_*ij*_(*t*) over each of a chosen set of time windows (indexed by *r*), as depicted schematically in Fig 1D. Here, we use eight time windows of 50 time steps each. These time-dependent synchronization matrices are used by the community detection algorithm to identify dynamic communities, as in Fig 1E. We compare the detected community structure to the underlying “ground truth” of the influence matrix (Fig 1F) to quantify the performance of the community detection algorithm under various conditions.

For each arrangement of underlying communities, we generate an ensemble of 20 influence matrices, all using the same intra- and inter-community connection probabilities (*P*_*in*_ and *P*_*out*_). We perform the dynamic simulation and community detection procedure separately on each network instance in the ensemble, resulting in a distribution of performance metrics. This distribution provides an estimate of the variance in community detection performance across networks with the same underlying structure, but noise or other variation affecting the existence or the measurement of some connections.

### Functional brain networks from fMRI Data

To demonstrate their utility in large-scale human brain networks, we apply these community detection and node removal techniques to two distinct functional brain network data sets, derived from two separate fMRI experiments on different groups of participants. We refer to these two functional data sets as the “single-task” and the “multi-task” data sets. Both experiments require participants to perform cognitive tasks during successive fMRI runs, but they provide a contrast between a set of behaviorally similar functional runs (“single-task” experiment) and a set of runs designed to elicit distinct cognitive functions (“multi-task” experiment).

Informed written consent was obtained from each participant prior to experimental sessions, and all procedures were approved by the University of California, Santa Barbara Human Participants Committee.

#### Experimental procedure

Single-task experiment: 126 healthy adult participants were scanned while performing a recognition memory task with lexical stimuli. During each of three identically designed functional runs, both previously examined and novel words were shown, and participants were required to distinguish between them with the aid of probabilistic cues. Each run was approximately 8.5 minutes long. Due to various sources of attrition and technical issues, data from 22 participants was excluded, leading to a final analysis of 104 participants. For additional experimental details, see [8] and [22].

Multi-task experiment: Functional MRI data were collected from 116 healthy adult participants during a set of distinct cognitive states. Participants were scanned at rest (task-free) and while engaging in three functional tasks: an attention-demanding task, a memory task with lexical stimuli similar to that used in the single-task experiment, and another similar memory task with face stimuli. Due to various sources of attrition, only 77 participants are included in the final analysis. For more experimental details, see [23, 24], and [25].

#### Functional brain networks

A dynamic network is constructed separately from each participant’s measured functional activity. Each network contains *N* nodes, corresponding to the *N* = 194 brain regions of a “hybrid” anatomical atlas, an adaptation of the multi-resolution Lausanne2008 atlas minimizing variability in region size [23, 26]. This atlas was registered to MNI space for each participant. The same set of brain regions is used for all participants, and the brain regions do not change over time. Region-specific time series from each functional run were generated for each node by averaging the BOLD signal time series across all voxels within the brain region [23].

Each network has *E* = *N*(*N* − 1)/2 edges, each with a real-valued, non-negative connection weight that may change over time, taking a new value in each of *T* sequential time windows spanning the experiment. The weight of an edge between nodes *i* and *j* in a given time window *t*, denoted *A*_*ijt*_, is defined as the mean low-frequency (0.06-0.125 Hz) wavelet coherence between the BOLD time series of *i* and *j* within that time window [8, 27–30].

#### Community detection

For each participant’s dynamic functional network, the community detection method described above is applied to find the partition that maximizes the multislice modularity *Q*. In these brain networks, the spatial resolution parameter *γ* is chosen with the analysis described in [8]. The most informative spatial scale is expected to be the one giving the most consistent community partitions across randomly seeded stochastic runs of the locally greedy modularity maximization algorithm. Following this reasoning, community detection is performed independently across a range of *γ* values, and the mean z-score of the Rand index between each pair of partitions generated by 100 algorithm runs at each *γ* value is computed, providing a quantitative measure of similarity across the partitions [31]. The Rand z-score is chosen because it inherently provides a comparison to a null distribution that takes the number and size of communities in each partition into account; it can be calculated analytically; and its behavior in the context of modularity maximization on functional brain networks has been previously characterized [11, 31]. The optimal *γ* value is that giving the highest average Rand z-score across pairs of algorithm runs and across participants, indicating the most consistent community partitions. When there is no clear choice (i.e., when the *γ* landscape is relatively flat), a near-optimal value is chosen based upon the expected number of functional communities [8]. After choosing a spatial scale, a temporal scale (*ω* value) is determined by choosing the value that maximizes the variance in flexibility across network nodes, where a node’s flexibility measures the number of times it switches community assignments between adjacent time windows (see Eq 2). This ensures that the algorithm will resolve high-flexibility nodes from those that remain within the same community throughout the experiment. See Discussion for further treatment of resolution parameters.

#### Comparison to known functional systems

In the absence of a clear “ground truth” benchmark for human functional brain networks, precise evaluation of community detection performance is a challenge. We use a basic partition of functional systems within the brain to assess whether these methods resolve brain systems with different functional roles from each other [4]. In this partition, each of the 194 brain regions is assigned to one of ten systems: auditory, cingulo-opercular, default mode, dorsal attention, fronto-parietal, somatosensory, subcortical, ventral attention, visual, and other. These systems were identified with a network-based clustering approach [4], and have been used to describe and quantify system-specific functional brain interactions [6, 8, 27, 32–34]. The assignment of regions to systems used here is based upon the primary functional roles of different anatomical brain areas, as detailed in [33] and [27].

We use *recruitment* to quantify the relationship between known functional systems and the communities detected in the data. The *node-specific recruitment* of a brain region is a measure of the consistency with which that region is assigned to the same community as other nodes in its own functional system. It is given by

$$\begin{array}{c} R\left(i\right)=\frac{1}{n(s_{i})-1}\sum_{j\neq i}\delta \left(c_{i},c_{j}\right)\delta \left(s_{i},s_{j}\right). \end{array}$$ (7)

Here, *s*_*i*_ denotes the functional system of brain region *i*, *n*(*s*_*i*_) gives the total number of regions in system *s*_*i*_, and *c*_*i*_ denotes the community assignment given to *i* by the community detection algorithm. The Kronecker deltas *δ*(*s*_*i*_, *s*_*j*_) and *δ*(*c*_*i*_, *c*_*j*_) count the region pairs (*i*, *j*) that belong to both the same known functional system and the same data-driven community. Thus, a brain region will have a high recruitment coefficient if the data-driven community to which it is assigned also contains a high fraction of functionally similar nodes.

We define the *system-specific* or *system recruitment* *Ψ* of a given system *S* as the average node-based recruitment of all nodes in the system, given by

$$\begin{array}{c} \Psi \left(S\right)=\frac{1}{n(S)(n(S)-1)}\sum_{ij,i\neq j}\delta \left(c_{i},c_{j}\right)\delta \left(s_{i},S\right)\delta \left(s_{j},S\right). \end{array}$$ (8)

This measures the extent to which nodes in system *S* are cohesively grouped together in the same community.

These recruitment metrics are inspired by similar quantities used in Refs. [27] and [35], but have been adapted to allow comparison of the metrics across scanning runs and subjects, and to avoid self-comparisons between nodes.

This basic partition into functional systems enables a quantitative assessment of the overlap between detected communities and the broad functional organization of the human brain. However, it does not fulfill the function of the “ground truth” benchmarks used in the synthetic oscillator networks. In particular, any “true” underlying functional modules driving the measured brain dynamics in these fMRI experiments would be expected to change dynamically as different brain systems are recruited for different cognitive tasks. This partition also does not capture the likely individual, temporal, and situational variation in the organization of brain function within a single task.

## Results

### Community detection in synthetic networks

Here, we demonstrate the performance of the modularity maximization community detection technique on a set of synthetic Kuramoto oscillator networks, detecting communities both before and after the targeted removal of nodes, and assessing the effect on performance.

As described in Methods, the basic system studied is a network of *N* oscillators, each with its own intrinsic frequency *ω*_*i*_, as well as influences from the other oscillators which are described by a time-independent binary influence matrix *C*. We simulate the network dynamics resulting from this underlying pattern of influence, and track the observed synchronization dynamics that result. These synchronization networks serve as a simplified analogy to functional brain networks, reflecting both direct and indirect influence among network nodes through a measurement of observed coherence [21]. By performing community detection on the observed synchronization dynamics of Kuramoto networks, we determine the accuracy with which these techniques can uncover the true underlying network communities in the presence of inherently multi-scale dynamics.

#### Comparing single-scale and multi-scale networks

We begin by investigating single-scale networks with communities of a single size. The first of these is a network of *N* = 60 nodes, containing three underlying communities of 20 nodes each. This modular pattern of underlying influence between oscillators is captured in the influence matrix *C*, which is generated randomly for each of an ensemble of 20 instances of the network, according to the relevant *P*_*in*_ and *P*_*out*_ probabilities. The mean of *C* over this ensemble is depicted for this network in Fig 3A, in which the three underlying communities of influence are clearly visible in a block-diagonal arrangement.

The dynamics of the Kuramoto network obeying these influences is then numerically simulated by solving Eq 5, and the average synchronization is calculated across each of eight separate time windows of 50 time steps each. Fig 3B shows an example synchronization matrix from a single time window in a single network instance. Here, although node pairs from the same underlying community are more synchronized than those from different communities on average, the delineation between communities appears more ambiguous than in the underlying influence matrix from panel A.

The community detection algorithm is then applied to the observed synchronization, for each of the 20 network instances in the ensemble. The performance is summarized in Fig 3C, the detection probability matrix *D*, in which each entry *D*_*ij*_ gives the percentage of instances in the ensemble in which nodes *i* and *j* are identified as belonging to the same community. (This matrix is averaged over the eight 50-step time windows for each simulation, since the results are very similar across time windows.) The shapes of the underlying communities from the influence matrix are evident here, indicating that the algorithm can detect the underlying communities on average over the whole ensemble. However, the detection probability for individual node pairs is usually less than 1, meaning that in individual network instances, the algorithm often misses the correct underlying influences.

We compare this performance to that of the same algorithm on a network of (*N* = 40 nodes), consisting of five underlying communities of 8 nodes each. Fig 3D, 3E and 3F describe this network in an manner analogous to the previous panels. The average of the binary influence matrix across all networks in the ensemble (Fig 3D) again shows a clear delineation of the underlying communities. An example of the synchronization dynamics of the network (Fig 3E) shows that the dynamics between node pairs within these smaller communities tend to be more strongly synchronized than within the larger communities from Fig 3B. The detection probability matrix in Fig 3F indicates that the algorithm detects almost all of the node pairs that share the same underlying community, but also falsely classifies some node pairs as having an underlying influence on each other.

Next, we compare the single-scale networks to the multi-scale network described above, consisting of three 20-node communities and five 8-node communities on underlying influence. Fig 3G, 3H and 3I show the binary influence matrix, the synchronization dynamics, and the detection probability matrix for this network, respectively. While the influence matrix and synchronization dynamics look relatively similar in the multi-scale network to the corresponding single-scale networks, the detection probability matrix is notably different. Many false underlying influences are detected, with the algorithm displaying particularly poor performance in distinguishing different communities of the same size from each other.

Fig 3J summarizes the changes in community detection performance between single- and multi-scale networks, by plotting the true positive rate for detection of node co-assignments against the false positive rate. For each ensemble of networks, the results from the 20 instances are shown as individual dots, highlighted by an ellipse with its center at the mean and its semimajor and semiminor axes illustrating the standard deviation of the ensemble results. An instance with perfect detection would fall in the upper left corner (*TPR* = 1 and *FPR* = 0), finding 100% of existing connections and no false positives.

The unfilled dots show performance on single-scale networks, with each representing one instance of a single-scale network with 20-node communities (blue) or 8-node communities (orange). While the community detection algorithm shows low false positive rates on both of these networks, on the 20-node network it displays significantly lower true positive rate, meaning it tends to split up the “true” communities and identify them as smaller subsets.

The filled dots show performance on multi-scale networks. This figure only shows the classification performance on node pairs where both nodes belong to communities of 20 nodes (blue) or where both belong to communities of 8 nodes (orange). The results measure how well the algorithm distinguishes communities *of the same size* from each other, without considering its ability to distinguish between communities of different sizes. This allows direct comparison of results between single-and multi-scale networks for each community size. The algorithm shows a slightly better true positive rate on the 20-node communities within the full multi-scale network, compared to in a single-scale network. However, this comes at a cost of a substantially higher false positive rate. In addition, the detection of 8-node communities deteriorates in a multi-scale network. The algorithm tends to combine multiple 8-node communities into one, producing more false positives than in the single-scale network.

#### Node removal

We now demonstrate community detection performance under removal of *a priori*-identified groups of nodes, as schematically represented in Fig 2. From the observed network dynamics, the synchronization matrix is computed while ignoring the removed nodes, creating a new network that includes no direct information about the removed nodes or their connections to the remaining nodes. The community detection method is then applied to these smaller functional networks to produce community assignments for all the remaining nodes.

First, we remove the larger communities and test the effect on the ability of the algorithm to identify the smaller communities. Note that in terms of dynamics, the larger communities are also consistently less synchronized compared to the smaller ones. Fig 4 shows average influence matrices (A-D) and detection probability matrices (E-H) for the full multi-scale network, compared to the network with one, two, and three of the 20-node communities removed. Fig 4I summarizes the changing community detection performance (computed for node pairs in 8-node communities only) as progressively more large communities are removed. Dots indicate performance on individual network instances, with colors distinguishing network ensembles with different numbers of nodes removed, and shaded ellipses highlighting the means and standard deviations of the different ensembles. With one (yellow) or two (red) large communities removed, there is no significant difference in true or false positive rates from the full multi-scale network, shown in orange (one-sample t-test, *p* > 0.1). However, with all three large communities removed (purple), the false-positive rate distribution is significantly different from all three other ensembles, and the true-positive rate distribution is significantly different from the full network ensemble and the ensemble with two large communities removed (t-test, *p* < 0.05, Bonferroni corrected for multiple comparisons).

Next, we remove the smaller, more synchronized communities, and measure the effect on the ability of the algorithm ability to identify the larger, less synchronized communities. Here, the successive removal of the smaller but more coherently connected 8-node communities substantially improves the ability of the community detection algorithm to distinguish the 20-node communities from each other, as shown in Fig 5. Each successive removal decreases false positive rates and increases true positive rates on average. All three distributions differ significantly in false positive rate, and the distribution with all smaller communities removed differs in true positive rate from the full multi-scale network (t-test, *p* < 0.05, Bonferroni corrected for multiple comparisons).

Note that although smaller, the 8-node communities are more cohesively connected overall, tend to be more synchronized, and are substantially easier for the algorithm to identify than the larger communities, both in single-scale networks and in the full multi-scale network. This may reflect that the resolution parameters used, *γ* = 1 and *ω* = 1, are more well-suited to detecting communities at the 8-node scale than the 20-node scale, given the dynamics in these networks.

Overall, we find that for the smaller, more synchronized 8-node communities, the choice of resolution parameter in the context of the other network parameters and dynamics allows relatively clean detection in both the original single-scale network and the full multi-scale network, although the multi-scale network causes an increase in false positive identifications. When the 20-node communities are removed from the multi-scale network, the false positive rates for the 8-node communities drop again, but the performance does not match that in the single-scale network (Fig 3J).

The larger and less synchronized 20-node communities are detected with relatively low true positive and false positive rates when in single-scale networks, as the algorithm tends to “split” the larger communities and identify them as smaller subsets. When embedded in multi-scale networks with smaller and more synchronized communities, the larger communities are detected with a much higher false positive rate (and only a mild improvement in true positive rate), as the algorithm often groups the larger communities together into an even larger super-community. However, when the smaller 8-node communities are removed from the multi-scale network, the detection of the 20-node communities improves markedly in true positives, while false positives are eliminated almost entirely, and the three underlying communities are cleanly detected in most instances.

Taken as a whole, these results demonstrate that in a relatively simple dynamic system, with highly interconnected communities of influence and low noise, embedding communities within multi-scale networks makes them more difficult to identify than they are in single-scale networks. However, the removal of subsets of network nodes during the community detection process can also dramatically improve detection of the remaining network communities, particularly when removing nodes that belong to highly synchronized communities.

### Community detection and node removal in brain networks

We now apply these methods to human functional brain networks extracted from two distinct fMRI data sets. As described in Methods, the “single-task” data set involves the performance of a single cognitive memory task for three sequential scanning runs, while the “multi-task” data set records the activity during a set of four sequential cognitive tasks, including rest and three different memory- and attention-recruiting tasks. Thus, we expect these data sets to display fundamentally different dynamics.

#### Spatial and temporal community structure

In this section, we summarize basic characteristics of identified dynamic community structure in the two experiments, with all brain regions included in the analysis. Subsequently, we will consider how these spatial and temporal characteristics are altered by the targeted removal of nodes before community detection.

**Fig 6:**
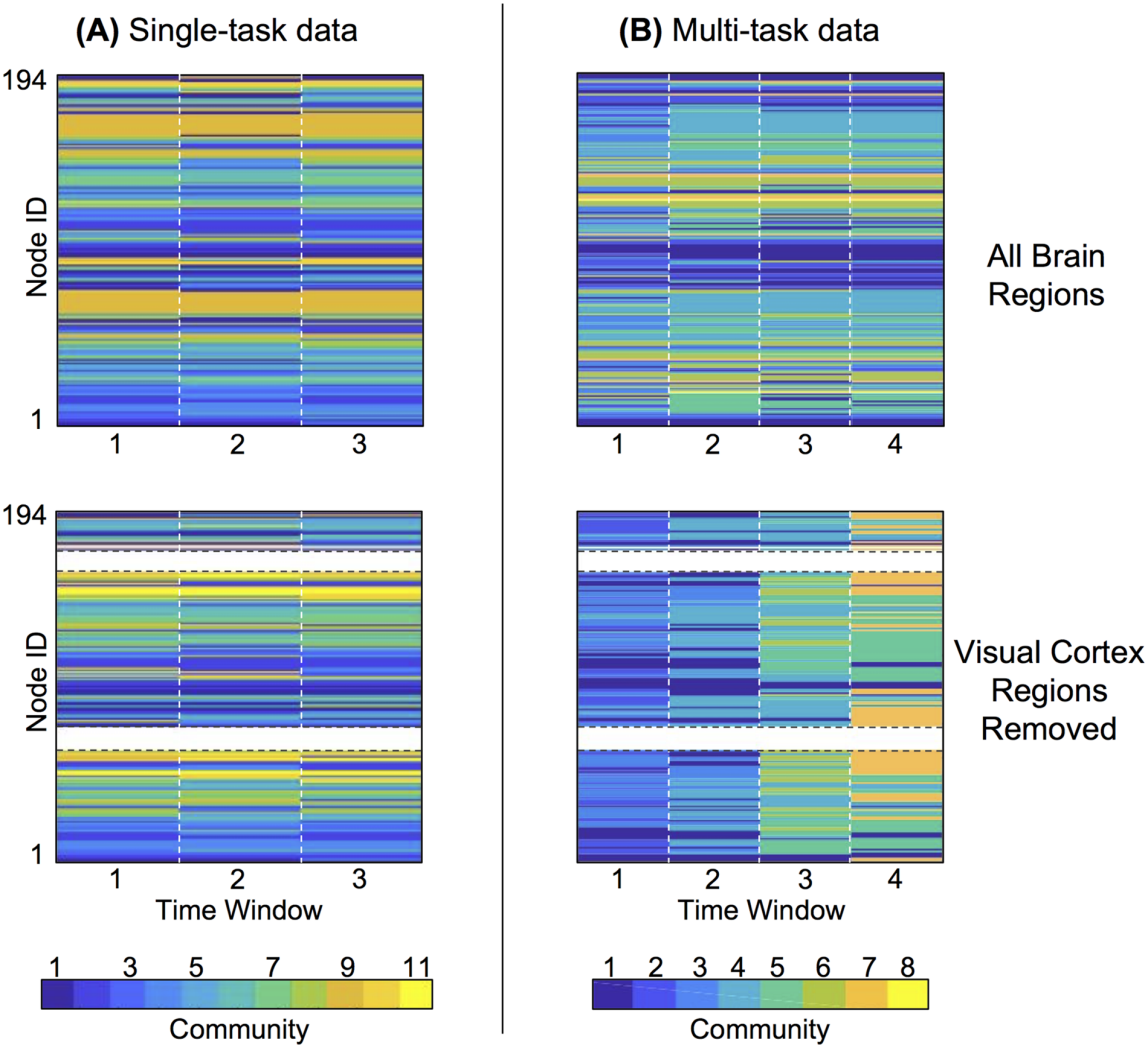
Node removal reveals dynamic changes in community structure in task-based functional brain networks. A: Visualization of community structure of a single participant during the single-task experiment. Each time window represents a single functional run during which the same lexical memory task is performed. The top panel shows community structure with all brain regions considered; visual cortex, which typically contains the least flexible nodes across functional runs, is visibly grouped into the temporally stable orange community (9). The bottom panel shows community structure with regions in the visual cortex removed from the functional network; these regions are now shown in white and have no community assignment. With the removal of vision nodes, the single-task experiment still shows largely temporally consistent communities. B: Community structure of a single participant in the multi-task experiment, in which each time window represents a single functional run containing a different cognitive task or a resting-state scan. Visual regions, which are explicitly required to perform tasks in time windows 2-4, again form the most stable community with the lowest flexibility (community 4) in the top panel. In the bottom panel, with visual regions removed, distinct differences are seen between the community structure in different tasks, including new communities arising as brain dynamics reconfigure for each new task.

In the single-task experiment, each participant has a *community number* between 7 and 24, with an average of 12.4. The community structure of one example participant in this experiment is visualized in the top panel of Fig 6A. In the multi-task experiment, each participant has between 5 and 12 dynamic communities, with an average of 6.9. The top panel of Fig 6B shows the community structure of a single example participant in this experiment.

We observe that for both experiments, brain regions in visual cortex tend to form the most consistently cohesive communities across across time windows, which represent separate functional runs within the experiments. For example, visual cortex regions form the clearly consistent orange community in the top panel of Fig 6A, and the corresponding teal community in time windows 2-4 of the top panel of Fig 6B. (Time window 1 in the multi-task experiment corresponds to the resting state scan, in which visual functions are not explicitly required.)

**Fig 7:**
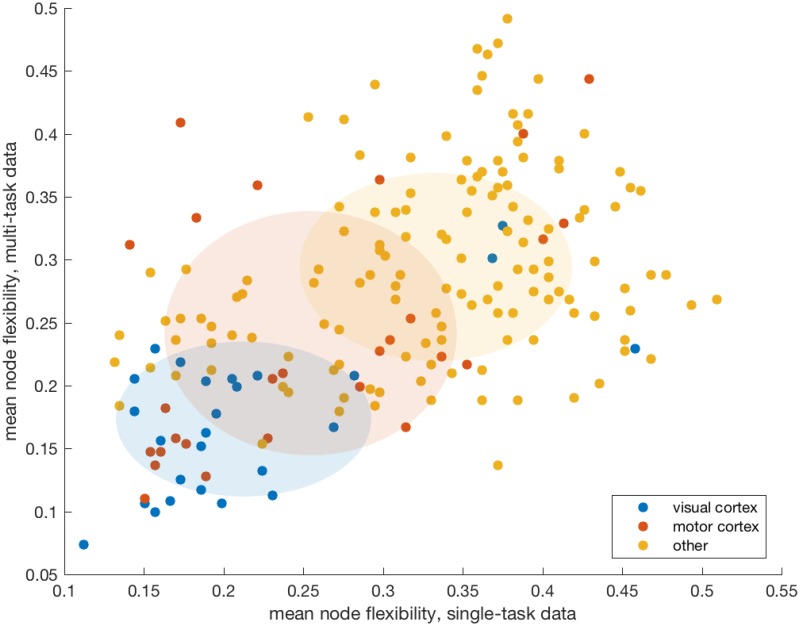
Region-specific flexibility. Average flexibilities of the 194 network nodes corresponding to brain regions. Each dot represents a single region/node, plotted according to its flexibility in switching between communities across runs of the single-task experiment (x-axis) and the multi-task experiment (y-axis). Flexibility is averaged over all participants in each study. There is a correlation between the flexibility of a brain region in the two different experiments. In particular, visual regions (blue circles) tend to be the least flexible.

We use the *flexibility* (Eq 2), which can range from 0 to 1, to quantitatively assess these observations on the consistency of community assignments. The average flexibility over all brain regions and participants is 0.31 for the single-task data, and 0.27 for the multi-task data. These are remarkably similar values given the distinctly different dynamic structure of the experiments, and the presence of task switching in the multi-task experiment only. Fig 7 shows the flexibility of each separate brain region, averaged across participants. As reported in [8], and consistent with observations in the example in Fig 6, the least flexible regions are largely found in visual and motor cortices. These areas are consistently recruited throughout all three functional runs of the single-task experiment, and runs 2-4 of the multi-task experiment, which require viewing a lexical stimulus and pressing a button to respond.

This similarity in flexibility distributions, despite the dynamic differences in the experiments, may result from the choice of a temporal resolution parameter that maximizes variation in flexibility across nodes. This effectively broadens the distribution in this direction and increases its likelihood of being centered. A Pearson correlation of *r* = 0.5 (*p* < 0.01) exists between the average flexibility of brain regions in the single-task experiment and the average flexibility of the same regions in the multi-task experiment.

#### Node removal from visual cortex

Both the single- and multi-task experiments strongly recruit visual brain regions, which consequently form strongly cohesive dynamic communities. However, the dynamic differences between the two experiments are not resolved by the community detection approach, and it is possible that the strength of the visual cortex masks the existence of other functional communities at different spatial scales, as in the synthetic oscillator networks. To determine whether better community resolution is possible in the non-visual brain regions, we repeat the community detection procedure after removing selected subsets of nodes, as shown schematically in Fig 2.

We remove the brain regions associated with vision, as classified according to [4]. The bottom panels of Fig 6A and 6B show the community detection results when the visual cortex regions are left out of the network, for a single example participant. The dynamic community structure in single-task data does not appear largely different. However, the multi-task dynamic community structure drastically shifts in this example; without the vision regions to provide a coherent community that persists across functional runs at this scale, rearrangements of brain regions in different task conditions are identified as entirely new communities. Since the resolution parameters in the algorithm are not re-scaled with the removal of vision nodes, these results provide a glimpse into the change in the relevant scales of brain network resolution across functional task conditions.

#### Resolution of known functional systems

To move beyond qualitative observation of a single example participant, we use the *recruitment coefficient* to quantify the extent to which targeted removal of visual nodes improves the resolution of other functional brain systems.

**Fig 8:**
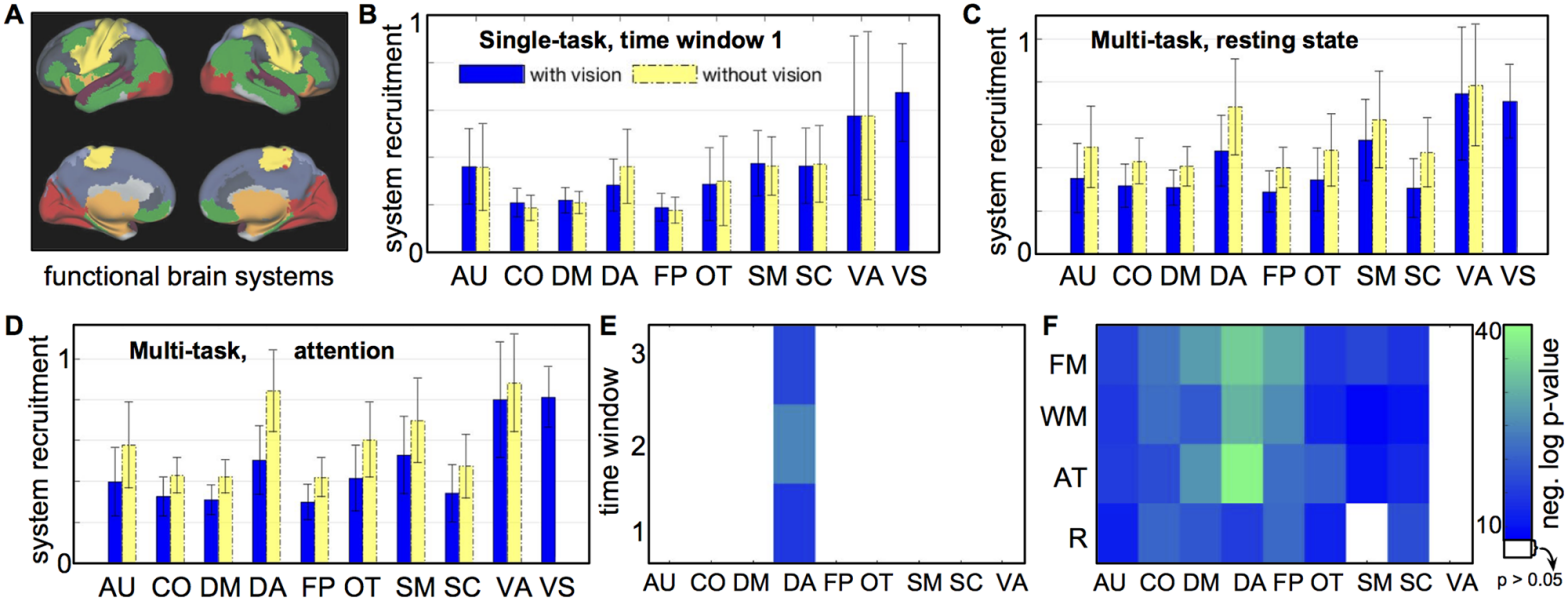
Effect of targeted node removal on resolution of known functional systems. A: Location in the brain of the ten functional systems: auditory (AU), cingulo-opercular (CO), default mode (DM), dorsal attention (DA), fronto-parietal (FP), somatosensory (SM), subcortical (SC), ventral attention (VA), visual (VS), and other (OT). B: System-specific recruitment coefficients with (blue) and without (yellow) the targeted removal of visual cortex regions, for the first functional run of the single-task experiment. Colored bars show the mean and black error bars the standard deviation over participants in each experiment. C: System-specific recruitment coefficients, analogous to panel B, for the resting state portion of the multi-task experiment. D: System-specific recruitment coefficients for the attention-demanding portion of the multi-task experiment. (Results from all functional runs are displayed in S1 Fig.) E: Depiction of systems and time windows in which targeted removal of visual cortex regions leads to significant increase in system-specific recruitment in the single-task experiment (one-sided paired t-test, *p* < 0.05, Bonferroni corrected for multiple comparisons). F: Depiction of systems and tasks/time windows in the multi-task experiment with significant increase of system-specific recruitment after node removal. Tasks include resting state (R), attention (AT), word memory (WM), and face memory (FM). In E and F, colored entries indicate a significant increase, with the color corresponding to the level of significance (negative logarithm of corrected p-value). In the multi-task experiment, targeted node removal significantly increases recruitment in most functional systems. In the single-task experiment, this only occurs in the dorsal attention system.

For each participant, we quantify the extent to which the community detection resolves known functional systems by computing the recruitment coefficient for each brain region (Eq 7), and the mean *system-specific recruitment* (Eq 8) for each of the ten functional systems. Fig 8A depicts the locations of the ten functional systems in the brain. Fig 8B plots the system-specific recruitment for each of the ten systems, during the first functional run of the single-task experiment. Each bar denotes the mean of the system-specific recruitment over all participants, and the error bars indicate the standard deviation over participants. Blue bars show the recruitment for each functional system when community detection is performed on the entire brain network, while yellow bars show the recruitment when visual regions are removed from the community detection analysis. In the single-task data, node removal does not make a significant difference in the recruitment of most systems, but it does significantly raise recruitment in the dorsal attention system (see statistics in Fig 8E). This indicates that brain regions associated primarily with top-down attention are more consistently grouped together into the same community when vision is removed.

Fig 8C and 8D show system-specific recruitment results for two functional runs of the multi-task experiment: the resting state scan and the attention task scan, respectively. (Results from all time windows and tasks in both experiments are included in S1 Fig.) Here, the removal of visual cortex regions significantly increases recruitment in almost every remaining functional system. The only exceptions are the ventral attention system in both cognitive states, and the sensory-motor system in the resting state (see details in Fig 8F). This indicates that in the multi-task experiment, targeted removal of the highly coherent visual brain regions enables the community detection algorithm to better resolve the involvement of the remaining brain in several other broad functional roles.

#### Resolution of dynamic task structure

Having demonstrated that targeted removal of brain regions in the visual cortex during community detection improves spatial resolution of other functional systems, we use measures of flexibility to assess the effect of this targeted removal on the detection of task-switching dynamics.

**Fig 9:**
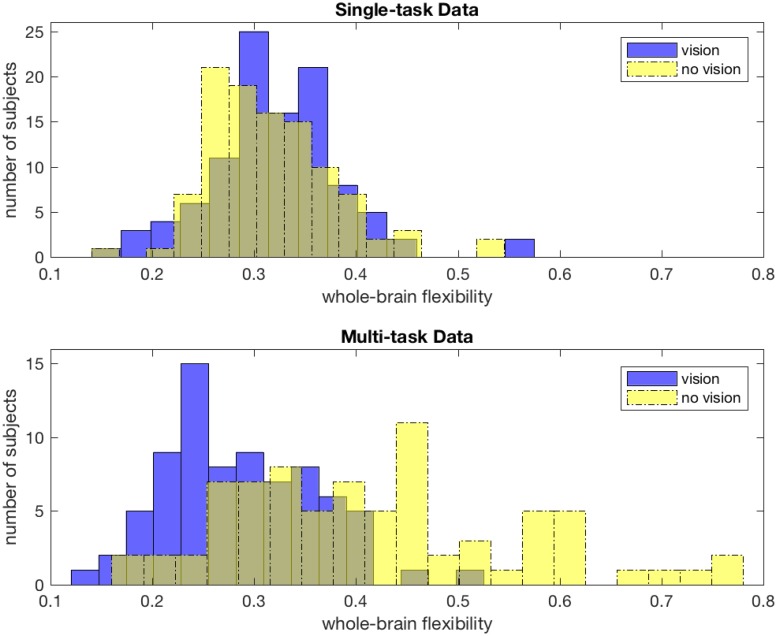
Effect of targeted node removal on resolution of task-switching dynamics. Whole-brain flexibility distributions as a result of dynamic community detection both with (dotted lines) and without (solid lines) brain regions from visual cortex, with single-task results in blue and multi-task results in red. Single-task distributions do not significantly change with removal of visual regions (paired t-test, *p* > 0.1), but multi-task distributions show a significant shift (paired t-test, *p* < 0.001).

For each participant, the *whole-brain flexibility* is computed by averaging the flexibility of all *N* = 194 brain regions to give a single measure. The distribution of whole-brain flexibility over participants is plotted in Fig 9, for community detection performed with all nodes (blue bars) and with the visual cortex nodes removed from the network (yellow bars). Single-task results are shown in the top panel, and multi-task data in the bottom panel. The flexibility distributions derived from all brain regions are similar between the two data sets, which is unsurprising given that the temporal resolution parameters were chosen independently to maximize variation in flexibility for each dataset. However, when visual regions are removed from the network, the differing properties of the two data sets become strongly evident. While the mean of the flexibility distribution across participants in the single-task experiment remains statistically indistinguishable before and after the removal of visual nodes (paired t-test, *p* > 0.1), the mean of the multi-task distribution shifts significantly (paired t-test, *p* < 0.001).

In this case, the removal of visual nodes distinguishes the community structure of these data sets beyond what can be observed through looking at the entire network. The upward shift in flexibility seen when visual nodes are removed from the multi-task dataset suggests that the strong connectivity and coherence within the visual cortex was the key source of temporal consistency in the originally detected communities. Without the consistently low-flexibility community of visual regions, the multi-task nature of this experiment becomes evident, as the community structure becomes better described by large differences between communities across tasks (as in the example in Fig 6B).

## Discussion

We introduce a technique of targeted node removal during dynamic community detection in complex networks, which can improve the resolution of community structure and dynamics. Using synthetic networks of Kuramoto oscillators, in which the underlying influences between nodes are well-defined, we quantify the performance of a common modularity-maximization community detection algorithm. We show that this algorithm can fail to resolve communities that occur at multiple spatial scales within the same network, compared to its detection of similar communities in single-scale networks. We also demonstrate that targeted removal of subsets of nodes, especially those that form the most functionally cohesive communities, can improve the resolution of communities among the remaining nodes.

It is important to note that the clusters of underlying influence in this synthetic network serve as a rough proxy for the basic functional organization that underlies the observed brain activity, but should not be interpreted as directly analogous to structural connectivity networks measured in the brain with diffusion MRI. The brain’s structural connectivity networks are sparse and heavily constrained by their spatial embedding [36], and are not necessarily expected to coincide with the results of community detection on dense functional connectivity networks measured with fMRI.

Instead, we hypothesize that community detection on functional connectivity matrices will provide information on a set of underlying functionally related areas which at least partially drive the observed activity patterns. In the synthetic oscillator networks, we interpret the influence matrix as a representation of this set of underlying functional groupings. In the brain, we use the known functional systems from Ref. [4], representing brain areas with shared functional roles, as a rough proxy for these underlying groupings.

In multi-scale networks like the human brain, it is unlikely that a single resolution parameter choice will elucidate the full community structure of interest, especially if the communities involved span widely disparate scales [10]. In many networks, there are conditions under which no parameter choice can fully distinguish the underlying communities, even when the communities of interest are of similar sizes [37]. In such situations, the choice of a single parameter may cause communities to be obscured. However, the targeted removal of node subsets that are strongly coherent can allow for clearer resolution of the communities formed by the remaining nodes.

To illustrate targeted node removal in a network neuroscience context, we apply it to functional networks derived from fMRI measurements of large-scale human brain activity. We use data from two distinct fMRI experiments: one involving repeated performance of a single task, and the other consisting of a succession of tasks designed to elicit differing cognitive functions. We expect the identified community structure in the single-task experiment to display consistency across functional runs as the same task is repeated, and that in the multi-task experiment to show rearrangement of communities over time in conjunction with task switching. However, when comparing the two experiments, the established algorithm alone finds no significant difference in the flexibility of brain regions between communities. It does, however, identify a strongly cohesive and inflexible community of nodes in the visual cortex. Upon removing the regions in the visual cortex during dynamic community detection, the ability of the algorithm to resolve certain other known functional systems within the brain significantly improves.

In the single-task experiment, removal of visual nodes significantly improves the recruitment coefficient of the dorsal attention system, composed of brain regions associated with the top-down directing of attention [38]. None of the other examined brain systems show significant improvement in the single-task experiment, possibly indicating that the presence of a strong visual community at this particular scale does not mask other functional dynamics in this experiment.

In the multi-task experiment, removal of visual nodes significantly improves the system-specific recruitment coefficient of almost every other functional system. This suggests that the coherence of the visual regions, especially as one of the few communities that is not expected to experience major shifts across different tasks, had significantly masked the functional coherence of several other functional modules in this experiment. The dorsal attention system again shows the most significant shift in recruitment coefficient, especially in the attention-related task, and to a lesser degree in the memory-related tasks, which also require directed attention. The ventral attention system, which is associated with responses to infrequent or unexpected cues [38], does not show a significant increase in recruitment; this may be related to the relatively small size of this brain system (4 regions).

Furthermore, upon removal of visual cortex regions, the dynamic differences between these experiments become evident. The average flexibility of brain regions in the multi-task experiment shifts sharply up, reflecting task switching, while the flexibility in the repeated single-task experiment stays the same. These results show that targeted node removal can not only improve the ability of the algorithm to resolve important dynamic changes in community structure, but also allow for meaningful comparisons between data sets through observation of the changes in identified community structure when specific nodes are removed.

The inability of the algorithm to identify the dynamic differences between experiments likely stems in part from the method of selecting the resolution parameters, especially the temporal resolution parameter *ω* in Eq 1. Having chosen a value for the spatial resolution *γ*, *ω* is chosen to maximize the variance in flexibility among nodes, as averaged across all participants in the experiment [3, 11]. This favors a broad distribution of flexibilities between 0 and 1, with an average likely to be nearer the center (0.5) than the extremes. Thus, a separate choice of *γ* and *ω* for each data set serves to effectively push all whole-network flexibilities toward a similar mean. This minimized differences in whole-network flexibility distributions between data sets, even if their nodes have relatively different flexibility patterns.

However, when the same nodes are removed from multiple networks, the algorithm responds differently if those nodes played different roles in the organization of the networks. As we see in the single-task and multi-task data sets, when highly coherent visual cortex regions are removed from the brain network during community detection, the algorithm is able to successfully resolve differences in community dynamics between the two experiments, recognizing the increased flexibility of brain regions in participants when switching between cognitive tasks.

Comparing community structures between networks from different participant demographics and experimental conditions is critical for understanding how these conditions shape brain organization, and how the brain’s organization affects them in turn. Our results demonstrate that even with commonly used heuristics for selecting resolution parameters, there is no guarantee that the resulting community structure will capture information that is comparable between data sets, especially when those data sets are based upon different experimental designs, participants, or imaging protocols. Without “ground truth” knowledge or an understanding of precisely how parameter choices affect the results, there is no clear way to establish an equivalence between parameters—and, by extension, community structures—for different data sets. In the neuroscience data studied here, it is only by performing a *relative* comparison of these two data sets, with and without a subset of regions removed, that the clear dynamic difference between the experiments comes into focus. This method provides a promising approach for performing such relative comparisons among other data sets, in order to resolve the dynamic roles of brain regions within functional networks.

### Methodological considerations

The Kuramoto oscillator simulations in this work include a “ground truth” community structure, specified by the influence matrix, which allows us to precisely evaluate the ability of the community detection algorithm to uncover the underlying clusters that fundamentally drive activity patterns. However, these synthetic networks serve as a relatively abstract and simplified model of brain network dynamics, and are limited in their ability to account for more complex features of brain function that are likely to affect community structure. For example, the synthetic influence matrix contains purely topological communities, in contrast to functional brain networks derived from fMRI data, whose connection weights are substantially influenced by the spatial proximity of brain regions. Although the effects of targeted node removal in the synthetic networks cannot be naïvely assumed to hold true in functional brain networks, our complementary analysis of networks derived from brain data provides evidence that the technique can be practically employed to improve resolution of known functional systems in the brain. It will be an important avenue for future research to develop and investigate synthetic networks that more realistically approximate the properties of functional brain networks, but remain straightforward to simulate and evaluate.

There are various possible strategies for targeted node removal, several of which have been previously proposed. We have focused on removing subsets of nodes defined by previous knowledge of their role in the system under study. For example, the fact that our experiments have visual stimuli provides good reason to expect the brain regions in visual cortex to be especially coherent, although they are not of particular interest to questions about memory and attention. Many fMRI experiments may have similar functional regions worth targeting for removal, or even multiple sets of regions that can be removed hierarchically.

Other strategies for choosing nodes to remove include data-driven removal, in which nodes are removed based on statistics of their network role and connectivity [39]; and model-guided removal, in which nodes are removed in order to optimize a model of underlying communities in the presence of noise [40]. Future work will quantify the performance of these methods, and assess their potential for identifying useful communities in human brain networks.

We have also focused our synthetic network experiments on multi-scale networks in which each node belongs to a single underlying community. This framework can be used in future studies to test community detection performance in more complex network configurations that are also likely to be relevant in network neuroscience applications. Examples include network nodes that belong to multiple communities, nodes that do not belong to any communities, and underlying communities of influence that change their organization over time.

This work is in part an attempt to better understand the behavior of community detection methods that have an inherent scale (often controllable by a resolution parameter), and to address resolution issues that may arise when such methods are applied to networks with varying community sizes, as is common in network neuroscience. It should be noted that since the modularity maximization method used here is subject to an inherent scale and a resolution limit that depends on the overall network size [19, 41], our proposed technique of removing highly coherent underlying modules from the network has the potential to reveal new communities among the remaining nodes that are not viable or meaningful communities in the context of the entire network.

We have demonstrated here that our technique works in practice to improve resolution of true underlying modules in synthetic networks, as well as known functional systems in brain networks, on which this modularity maximization method is widely used. We have also shown that our technique can provide important information about dynamics in temporal brain networks. However, recent literature suggests an alternative approach: the use of resolution-limit-free clustering methods, in which the optimal partitioning results for subgraphs are guaranteed to remain the same as in the full graph [41–43]. In will be important in future work to further investigate the behavior of these methods on temporal brain networks, including their ability to reveal underlying information about network dynamics on different time scales.

Finally, we note that we have not applied a threshold to our functional networks in advance of community detection. Weaker associations in functional human brain networks have been shown to contain functionally relevant and predictive information, and can feasibly occur even in strongly interconnected communities [44–46]. Our choice not to threshold retains this information, and avoids the issue of choosing a suitable threshold value with no ground truth information. However, retaining low-strength functional associations also increases the influence of noise in the network, which can impact the ability of clustering algorithms to identify communities cleanly. Several thresholding for brain networks have been explored [10, 47, 48]; in future work, it will be important to evaluate the effect on community detection performance of combining thresholding with the techniques proposed here.

## Supporting information

S1 Fig Effect of targeted node removal on resolution of known functional systems. System-specific recruitment coefficients with (blue) and without (yellow) the targeted removal of visual cortex regions, for the ten functional systems. Colored bars show the mean and black error bars the standard deviation over participants in each experiment. Panels A, B, and C show the three functional runs of the single-task experiment. All three runs consist of the same recognition memory task with lexical stimuli, and the runs are treated as three time windows in the dynamic functional brain networks. Panels D-G show the four time windows of the multi-task experiment, with each window encompassing a different task or cognitive state. These include resting state (D), an attention-demanding task (E), a recognition memory task with lexical stimuli (F), and a recognition memory task with face stimuli (G). H: Depiction of systems and time windows in which targeted removal of visual cortex regions leads to significant increase in system-specific recruitment in the single-task experiment. I: Depiction of systems and tasks (resting state (R), attention (AT), word memory (WM), and face memory (FM)) in the multi-task experiment with significant increase of system-specific recruitment after node removal. In H and I, colored entries indicate a significant increase (one-sided paired t-test), with the color corresponding to the level of significance (negative logarithm of Bonferroni-corrected p-value). H and I are reproductions of subfigures in the main manuscript.
(TIFF)
