# Supplementary figures and images for: Improving resolution of dynamic communities in human brain networks through targeted node removal
Source: PLoS One. 2017 Dec 20;12(12):e0187715. doi: 10.1371/journal.pone.0187715 (PMC5737970; doi:10.1371/journal.pone.0187715)

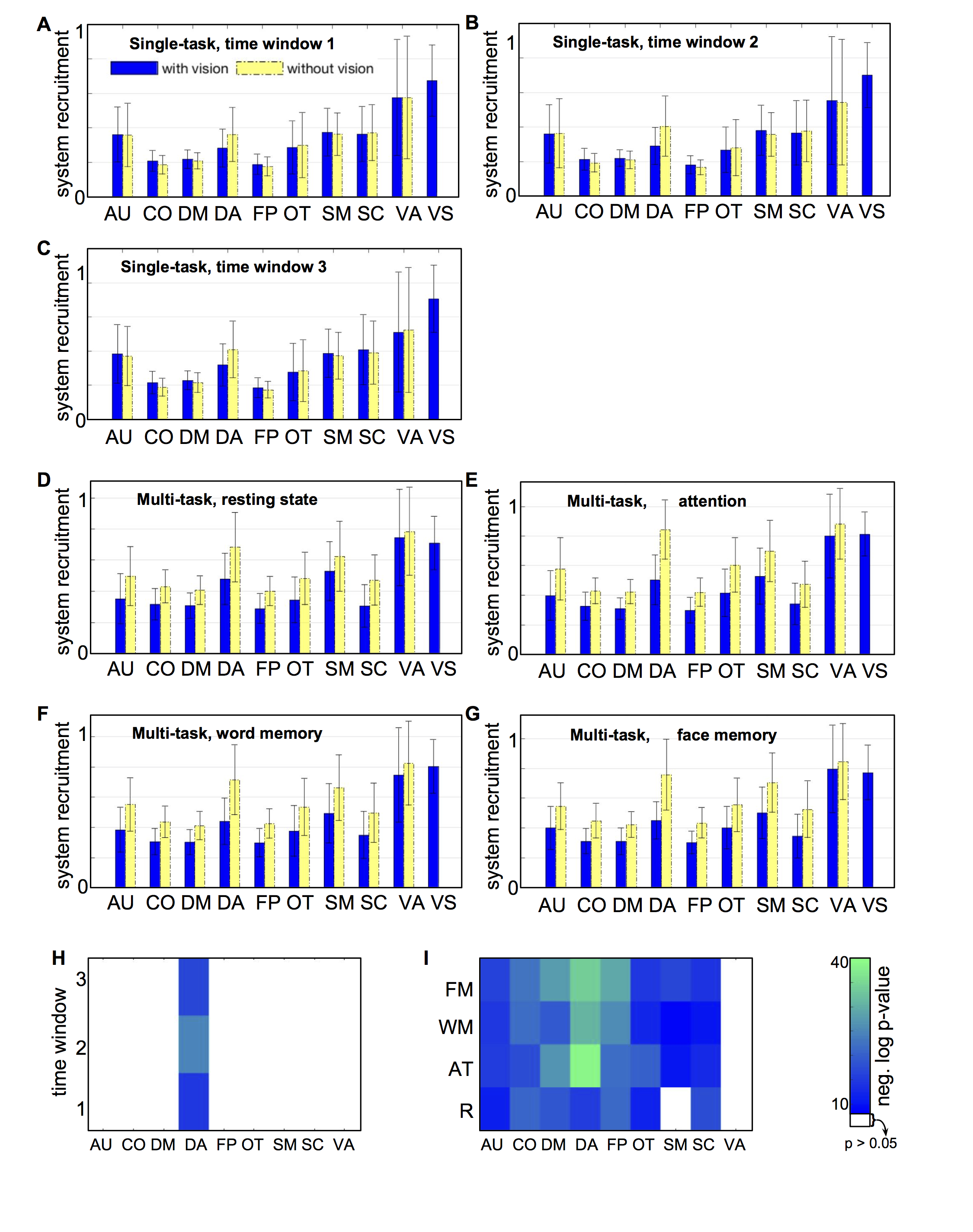

Supplement: S1 Fig — System-specific recruitment coefficients with (blue) and without (yellow) the targeted removal of visual cortex regions, for the ten functional systems. Colored bars show the mean and black error bars the standard deviation over participants in each experiment. Panels A, B, and C show the three functional runs of the single-task experiment. All three runs consist of the same recognition memory task with lexical stimuli, and the runs are treated as three time windows in the dynamic functional brain networks. Panels D-G show the four time windows of the multi-task experiment, with each window encompassing a different task or cognitive state. These include resting state (D), an attention-demanding task (E), a recognition memory task with lexical stimuli (F), and a recognition memory task with face stimuli (G). H: Depiction of systems and time windows in which targeted removal of visual cortex regions leads to significant increase in system-specific recruitment in the single-task experiment. I: Depiction of systems and tasks (resting state (R), attention (AT), word memory (WM), and face memory (FM)) in the multi-task experiment with significant increase of system-specific recruitment after node removal. In H and I, colored entries indicate a significant increase (one-sided paired t-test), with the color corresponding to the level of significance (negative logarithm of Bonferroni-corrected p-value). H and I are reproductions of subfigures in the main manuscript. (TIFF) [file pone.0187715.s001.tiff]
